# INTERGROWTH‐21st Birthweight Charts Offer Excellent Concordance With UK‐WHO Birthweight Charts Used by Neonatologists

**DOI:** 10.1111/1471-0528.18048

**Published:** 2025-01-20

**Authors:** Sophie Alexandra Relph, Julien Josseph Stirnemann, Raffaele Napolitano

**Affiliations:** ^1^ Fetal Medicine Unit University College London Hospitals NHS Foundation Trust London UK; ^2^ Elizabeth Garrett Anderson Institute for Women's Health University College London London UK; ^3^ Necker‐Enfants Malades University Hospital Paris France; ^4^ Paris Cit e University and IMAGINE Institute Paris France

There is a wide range of charts available for the assessment of estimated foetal weight (EFW) and measured neonatal weight centiles at a given gestational age. Charts vary in methodology, with descriptive and prescriptive, population and customised versions available, many designed with a high risk of bias [1, 2]. In many countries, including the UK, operators use varied charts for prenatal and neonatal care, leading to disagreement on diagnosis of abnormal growth. In determining which chart to apply in our tertiary centre and potentially nationwide, we aimed to identify a chart package that is applicable to our population, offers charts for assessment of EFW, actual birthweight and fundal height and is concordant with the UK‐WHO birthweight charts recommended nationally by the Royal College of Paediatrics and Child Health (RCPCH—rcpch.ac.uk) which are based on British growth reference centiles derived by Cole in 1990 [3]. Such concordance should facilitate agreement on small or large sizes, including the presence of growth restriction, between obstetricians and neonatologists, improving continuity of care with regard to the risk of perinatal morbidity and mortality. The INTERGROWTH‐21st package offers both foetal and neonatal charts and has previously shown good continuity of growth between prenatal and neonatal centiles at late pre‐term and term gestations in these populations when EFW in grams was calculated using both their formula including head and abdominal circumference only, or the Hadlock's formula, using three parameters (head, abdominal circumference and femur length) [4, 5]. INTERGROWTH‐21st prenatal chart centiles have also been shown to adequately fit the distribution of a large French prospective sample of ultrasound‐derived foetal measurements [6]. Here, we describe work to evaluate whether the INTERGROWTH‐21st birthweight centiles are concordant with those of the UK‐WHO birthweight charts for pre‐term and term births [7].

We used 10th, 50th and 90th centile thresholds published by the INTERGROWTH‐21st group for neonatal birthweight charts at each gestational day between 24 and 42 weeks to produce centile charts for male and female babies. UK‐WHO birthweight centiles have only been published in chart format, from which it is difficult to derive the exact birthweight for the 10th, 50th and 90th centiles during each gestational day. We therefore produced a simulation data set, including male and female neonates with birthweights of 10‐g increments between 400 and 5500 g, for each gestational day between 24 and 42 weeks. We calculated the UK‐WHO birthweight centiles using the Stata ‘zanthro’ command (StataCorp LLC, Texas, USA). *Zanthro* is a user‐written command that calculates z‐scores for anthropometric measures according to reference growth charts, with a specific option to apply the Cole UK‐WHO charts. We then converted z‐scores into birthweight centiles to identify the birthweight at the 10th, 50th and 90th centiles for each gestational age. We plotted all INTERGROWTH‐21st and UK‐WHO birthweight centiles onto the same chart, but separately for male and female babies.

In Figure 1, we show that the INTERGROWTH‐21st birthweight charts for male and female babies have excellent concordance with the UK‐WHO population reference charts used by UK neonatologists prior to 38 weeks and satisfactory concordance beyond this in terms of birthweight at each centile and overall trajectory. For males born at 38 weeks, the difference between charts is 10 g on the 10th centile, 60 g on the 50th centile and 80 g on the 90th centile (20, 30 and 50 g for females, respectively). By 42 weeks, this difference increases to 210 g on the 10th centile, 370 g on the 50th centile and 510 g on the 90th centile (200, 330 and 430 g for females, respectively). Differences in derived centiles for a given birthweight and gestational age by each chart are illustrated in Figure 1.

**FIGURE 1:**
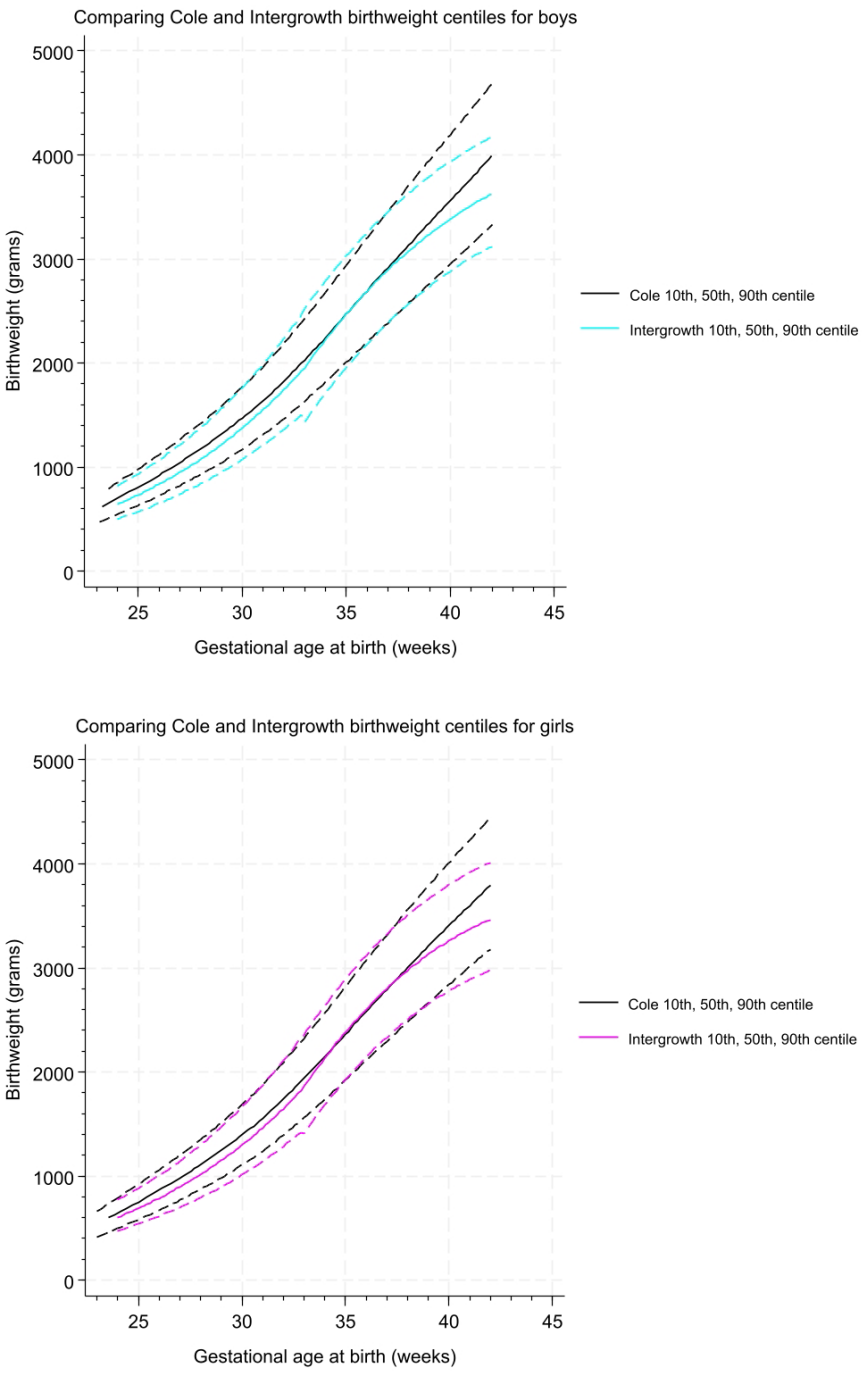
Birthweight size charts comparing Intergrowth‐21st and Cole et al. UK WHO birth weight centiles.

The study is limited by using a simulated data set to calculate the gestational‐age‐specific birthweight at each centile threshold; however, as the original formula has not been published by Cole et al., this was the most appropriate method to explore chart differences. We conclude that the INTERGROWTH‐21st birthweight charts satisfy our criteria for implementation in our UK maternity unit and nationally. INTERGROWTH‐21st provides a full package of charts for assessment of foetal and neonatal weights as well as fundal height charts (options not all available among UK‐WHO charts offering) with a satisfactory level of concordance with the UK‐WHO birthweight charts, nationally recommended by the RCPCH particularly for small for gestational age babies. Larger differences are seen for 50th and 90th centiles beyond 38 weeks gestation, with INTERGROWTH‐21st birthweight classifying a greater proportion of babies as large for gestational age.

## Author Contributions

R.S.A and N.R conceived the papr. R.S.A performed the analysis. All authors contributed to the design, analysis interpretation and writing.

## Conflicts of Interest

The authors declare no conflicts of interest.

## Data Availability

Data sharing is not applicable to this article as no new data were created or analyzed in this study.
